# Is there a role for regenerative medicine in chronic rhinosinusitis with nasal polyps?^☆^

**DOI:** 10.1016/j.bjorl.2016.10.002

**Published:** 2016-11-02

**Authors:** Fabiana C.P. Valera, Leandra M. Endam, Badr Ibrahim, Emmanuelle Brochiero, Martin Y. Desrosiers

**Affiliations:** a Centre de Recherche du Centre Hospitalier de l’Université de Montréal (CRCHUM), Montréal, Québec, Canada; b Universidade de São Paulo, Faculdade de Medicina de Ribeirão Preto (FMRP-USP), Divisão de Otorrinolaringologia, Ribeirão Preto, SP, Brazil; c Centre de Recherche du Centre Hospitalier de l’Université de Montréal (CRCHUM), Department of Otolaryngology, Montréal, Québec, Canada; d Université de Montréal, Department of Medicine, Montréal, Québec, Canada

During the past five years, over than 1600 articles have been published on the subject of Chronic Rhinosinusitis with Nasal Polyps (CRSwNP). Despite this, its physiopathology remains incompletely understood. CRSwNP is an overt and reverberant inflammatory disease, which leads to the recruitment of several inflammatory cells, characterized by the presence of eosinophils, neutrophils, and mast cells.^1^ Although a Th2-driven response was initially thought to be the principal inflammatory mechanisms present in CRSwNP, it is now recognized that both Th1 and Th2 responses can be observed in patients with CRSwNP, depending on ethnicity or on associated diseases.^1^ As example, Asian patients and those with cystic fibrosis tend to present more of a Th1-skewed inflammatory profile as compared to European nasal polyps. It should be noted that the reason for such an intense inflammatory process, and the different inflammatory patterns is still poorly understood.

An increased appreciation of the potential role of the nasal epithelium and its associated barrier and defensive functions may help improve our insights into CRS pathogenesis. Emerging evidence^1^, ^2^, ^3^ increasingly documents that nasal epithelial cells are not simply passive barriers between host interior and exterior environments, but instead play an active role in modulating and coordinating host responses to the external environment. They are capable of monitoring the environment for threats via pathogen detection, and can mount defensive responses by inducing signaling pathways, with generation of subsequent inflammation and cellular recruitment.

The integrity of the epithelial barrier is determined by tight junctions (located more apically, and composed of occluding, junctional adhesion molecule A and the claudin family), adherens junctions (composed by E-cadherin and catenins) and desmosomes (composed mostly by desmoglein and desmocollin).^2^ All these molecules regulate cell-to-cell permeability, and are associated with different pathways, with distinct roles in homeostasis.

An impaired epithelial barrier could be a rational cause for a reverberant process since increased permeability to allergens, pollutants, bacteria, viruses, fungi, and others^4^ would facilitate their penetration through into the superficial layer. In addition, this could contribute to dysregulated ion and fluid transport through epithelial cells as well as altered cellular function. In support of this, disrupted epithelia have now been proven to promote a Th2 signaling at lower airways,^4^ and asthma, chronic rhinosinusitis and, more recently allergic rhinitis^2^ have recently been associated with lower expression of tight junction proteins. Intriguingly, these disorders are also share similar genetic underpinnings.^5^

However, little is known about the potential causes for this barrier defects and its real impact on CRSwNP. Do epithelial defects lead to inflammation, or is the contrary, with environmental agents contributing to dysfunctional epithelium?

Our group^6^, ^7^ has previously performed pooled Genome-Wide Association Study (GWAS) to evaluate possible genetic polymorphisms associated with CRS. Among the potential 400 genes identified, the most importantly related ones were *LAMA2* (laminin-α2) and *LAMB1* (laminin-β1). Laminins are essential proteins at basal lamina, thus this result suggests that genetic disturbance(s) in cellular components may lead to epithelial dysfunction in CRS.

Conversely, other studies have demonstrated that the epithelial barrier could be compromised by extrinsic factors. In particular, the observed inflammation in CRS may contribute to epithelial dysfunction. Wise et al.^3^ showed that primary nasal epithelial cells, when exposed to IL-4 and IL-13, presented lower expression of JAM-A (junctional adhesion molecule A) and E-cadherin, with consequent diminished transepithelial resistance. A similar pattern was observed by Ramezanpour et al.^8^ after primary nasal epithelial cells were exposed to Th17 cytokines (IL-17, IL-22, and IL-26).

Bacterial factors may also play a role. Our group has demonstrated that primary nasal epithelial cells, when exposed to *Pseudomonas aeruginosa* diffuse material (PsaDM), exhibited a lower capability to repair wounds following injury, and also manifest a significant reduction in cystic fibrosis transmembrane conductance regulator (CFTR) expression and function, even in non-cystic fibrosis patients.^9^, ^10^ This finding is especially important after the description, also by our group,^11^ that inhibiting CFTR in non-CF nasal polyps significantly inhibited wound closure.

These inflammatory and infectious components may not only affect mature, differentiated cells but may also impact epithelial progenitor cells, thus interfering with epithelial regeneration and reparative processes in response to injury. Yu et al.^12^ demonstrated that epithelial basal cells from nasal polyps grow and proliferate slower than control mucosa, when both are submitted to the same experimental conditions. Moreover, in our own work, we observed that undifferentiated basal cell cultures raised from patients with CRSwNP (without CF) exhibited lower wound healing rates than controls (unpublished data).

All those findings suggest that epithelial repair is impaired in patients with CRSwNP and that regenerative medicine in this disease has been poorly explored until now. Enhanced epithelial repair could interfere with the reverberant inflammatory pattern present in patients with chronic rhinosinusitis, thus allowing disease resolution. This suggests it may represent an interesting novel therapeutic target, which could be addressed irrespectively of whether the cause was genetic or induced by external agents. While most researches on regenerative medicine today have focused on the use of stem cells,^13^, ^14^ other therapeutic approaches aimed at improving epithelial regeneration and repair may offer a benefit in treating CRSwNP, either as an isolated therapy, or concomitantly with current anti-bacterial and immunomodulatory strategies.

Continued efforts to better understand the mechanisms of wound healing and epithelial repair/regeneration in CRSwNP may thus yield novel therapeutic approaches for CRSwNP. By targeting this novel element of CRS pathogenesis which has until now not been addressed, we may usher in a new era in CRS treatment, thus improving outcomes for our CRS patients.

## Conflicts of interest

The authors declare no conflicts of interest.

## References

[1] Stevens W.W., Lee R.J., Schleimer R.P., Cohen N.A. (2015). Chronic rhinosinusitis pathogenesis. J Allergy Clin Immunol.

[2] Steelant B., Farré R., Wawrzyniak P., Belmans J., Dekimpe E., Vanheel H. (2016). Impaired barrier function in patients with house dust mite-induced allergic rhinitis is accompanied by decreased occluding and zoluna occludens-1 expression. J Allergy Clin Immunol.

[3] Wise S.K., Laury A.M., Katz E.H., Den Beste K.A., Parkos C.A., Nusrat A. (2015). IL-4 and IL-13 compromise the sinonasal epithelial barrier and perturb intercellular junction protein expression. Int Forum Allergy Rhinol.

[4] Georas S.N., Rezaee F. (2014). Epithelial barrier function: at the front line of asthma immunology and allergic airways inflammation. J Allergy Clin Immunol.

[5] Gupta J., Johansson E., Bernstein J.A., Chakraborty R., Khurana Hershey G.K., Rothenberg M.E. (2016). Resolving the etiology of atopic disorders by using genetic analysis of racial ancestry. J Allergy Clin Immunol.

[6] Mfuna-Endam L., Zhang Y., Desrosiers M.Y. (2011). Genetics of rhinosinusitis. Curr Allergy Asthma Rep.

[7] Bossé Y., Bacot F., Montpetit A., Rung J., Qu H.Q., Engert J.C. (2009). Identification of susceptibility genes for complex diseases using pooling-based genome-wide association scans. Hum Genet.

[8] Ramezanpour M., Moraitis S., Smith J.L.P., Wormald P.J., Vreugde S. (2016). TH17 cytokines disrupt the airway mucosal barrier in chronic rhinosinusitis. Mediat Inflamm.

[9] Trinh N.T.N., Bilodeau C., Maillé E., Ruffin M., Quintal M.C., Desrosiers M.Y. (2015). Deleterious impact of *Pseudomonas aeruginosa* on cystic fibrosis transmembrane conductance regulator function and rescue in airways epithelial cells. Eur Respir J.

[10] Ruffin M., Vilodeau C., Maillé É., LaFayette S.L., McKay G.A., Trinh N.T. (2016). Quorum-sensing inhibition abrogates the deleterious impact of *Pseudomonas aeruginosa* on airway epithelial repair. FASEB J.

[11] Trinh N.T.N., Bardou O., Privé A., Maillé E., Adam D., Lingée S. (2012). Improvement of defective cystic fibrosis airways epithelial wound repair after CFTR rescue. Eur Respir J.

[12] Yu X.M., Li C.W., Chao S.S., Li Y.Y., Yan Y., Zhao X.N. (2014). Reduced growth and proliferation dynamics of nasal epithelial stem/progenitor cells in nasal polyps *in vitro*. Sci Rep.

[13] Rosenthal N., Badylak S. (2016). Regenerative medicine: today's discoveries informing the future of medical practice. NPJ Regen Med.

[14] Pezato R., Gregorio L.C., Voegels R.L., Kosugi E.M., Pinna F., Perez-Novo C. (2016). Hypotheses about the potential role of mesenchymal stem cell on nasal polyposis: a soft inflamed tissue suffering from mechanical dysfunction. Austin Immunol.

